# Complete genomic sequences of *Propionibacterium freudenreichii* phages from Swiss cheese reveal greater diversity than *Cutibacterium* (formerly *Propionibacterium) acnes* phages

**DOI:** 10.1186/s12866-018-1159-y

**Published:** 2018-03-01

**Authors:** Lucy Cheng, Laura J. Marinelli, Noël Grosset, Sorel T. Fitz-Gibbon, Charles A. Bowman, Brian Q. Dang, Daniel A. Russell, Deborah Jacobs-Sera, Baochen Shi, Matteo Pellegrini, Jeff F. Miller, Michel Gautier, Graham F. Hatfull, Robert L. Modlin

**Affiliations:** 1 Division of Dermatology, Department of Medicine, David Geffen School of Medicine, University of California Los Angeles, Los Angeles, 90095 CA USA; 2 Department of Microbiology, Immunology and Molecular Genetics, David Geffen School of Medicine, University of California Los Angeles, Los Angeles, CA 90095 USA; 3 Equipe Microbiologie de l’œuf et des Ovoproduits (MICOV), Agrocampus Ouest, INRA, (UMR1253) Science et Technologie du Lait et de l’Œuf, Rennes, France; 4 Department of Molecular, Cell, and Developmental Biology, University of California Los Angeles, Los Angeles, CA 90095 USA; 5 Department of Biological Sciences, University of Pittsburgh, Pittsburgh, PA 15260 USA; 6 Department of Molecular and Medical Pharmacology, Crump Institute for Molecular Imaging, David Geffen School of Medicine, University of California Los Angeles, Los Angeles, CA 90095 USA; 7 California NanoSystems Institute, University of California Los Angeles, Los Angeles, CA 90095 USA

**Keywords:** *Propionibacterium freudenreichii*, Bacteriophage, Phage genomics, Cheese microbiota, *Cutibacterium acnes*

## Abstract

**Background:**

A remarkable exception to the large genetic diversity often observed for bacteriophages infecting a specific bacterial host was found for the *Cutibacterium acnes* (formerly *Propionibacterium acnes*) phages, which are highly homogeneous. Phages infecting the related species, which is also a member of the Propionibacteriaceae family, *Propionibacterium freudenreichii*, a bacterium used in production of Swiss-type cheeses, have also been described and are common contaminants of the cheese manufacturing process. However, little is known about their genetic composition and diversity.

**Results:**

We obtained seven independently isolated bacteriophages that infect *P. freudenreichii* from Swiss-type cheese samples, and determined their complete genome sequences. These data revealed that all seven phage isolates are of similar genomic length and GC% content, but their genomes are highly diverse, including genes encoding the capsid, tape measure, and tail proteins. In contrast to *C. acnes* phages, all *P. freudenreichii* phage genomes encode a putative integrase protein, suggesting they are capable of lysogenic growth. This is supported by the finding of related prophages in some *P. freudenreichii* strains. The seven phages could further be distinguished as belonging to two distinct genomic types, or ‘clusters’, based on nucleotide sequences, and host range analyses conducted on a collection of *P. freudenreichii* strains show a higher degree of host specificity than is observed for the *C. acnes* phages.

**Conclusions:**

Overall, our data demonstrate *P. freudenreichii* bacteriophages are distinct from *C. acnes* phages, as evidenced by their higher genetic diversity, potential for lysogenic growth, and more restricted host ranges. This suggests substantial differences in the evolution of these related species from the Propionibacteriaceae family and their phages, which is potentially related to their distinct environmental niches.

**Electronic supplementary material:**

The online version of this article (10.1186/s12866-018-1159-y) contains supplementary material, which is available to authorized users.

## Background

Most bacteriophage populations display a wide range of genetic diversity, including those phages infecting *Mycobacterium*, *Staphylococcus*, and *Pseudomonas* spp. [1–3]. In contrast, *Cutibacterium acnes* bacteriophages, whose host was formerly a member of the genus *Propionibacterium* and was recently reclassified [4], have been found to have a surprisingly limited genetic diversity [5]. Propionibacteria and Cutibacteria are Gram-positive, anaerobic to aerotolerant, rod-shaped bacteria of the Actinobacteria class, originally named for their unique metabolism, specifically the ability to synthesize propionic acid via the activity of transcarboxylase enzymes during fermentation [6]. *C. acnes,* along with *C. granulosum* and *C. avidum,* were formerly referred to as the cutaneous propionibacteria and are all members of the human skin microbiome. The classical type of propionibacteria, including *P. freudenreichii, P. acidifaciens*, *P. cyclohexanicum,* and *P. australiense,* have been found in milk and cheese, as well as in various other biotopes, such as silage, soil, pasture, and the human mouth [7, 8]. *C. acnes* and *P. freudenreichii* are not highly related at the nucleotide level, but show a high synteny with regards to their protein coding sequences [9]. *C. acnes* also has a lower average percent GC (60% vs. 67%) and encodes a larger proportion of genes involved in processes such as host association, tissue degradation, and iron acquisition [4, 9].

*P. freudenreichii* is specifically utilized in the manufacture of Swiss-type cheeses, such as Emmental, for ripening the culture. Through the Wood-Werkman cycle, this bacterium drives fermentation of lactate into acetate, propionate (which gives Swiss-type cheese its unique nutty and sweet flavor), and carbon dioxide (responsible for the holes or “eyes” in the cheese) (3 C_3_H_6_O_3_ → 2 C_2_H_5_CO_2_ + C_2_H_3_O_2_ + CO_2_) [10]. An estimated one billion living cells of *P. freudenreichii* are present in one gram of Emmental cheese [11]. In contrast to most lactic acid bacteria, this bacterium mainly breaks down lipids, forming free fatty acids. Recent research has focused on the possible probiotic benefits incurred from consuming *P. freudenreichii*. Notably, these bacteria have anti-inflammatory and probiotic properties for the gastrointestinal tract in animals [12] and have also been found to display cytotoxic activity against colon cancer cell lines in vitro [13, 14].

Bacteriophages that infect *P. freudenreichii* have been isolated from Swiss-type cheese [15, 16] and are common contaminants of the cheese manufacturing process. The genome of a single filamentous variant of *P. freudenreichii* phage has been described [17], the first filamentous phages of any Gram-positive bacterium to be reported. Most *P. freudenreichii* phages, however, are of the *Siphoviridae* type [15, 18], similar to the *C. acnes* phages. Recently, the complete genomes of two dsDNA tailed *P. freudenreichii* phages were reported [19]. These are distinct from the *C. acnes* phages but highly similar to one another, raising the question as to whether this group of phages also displays restricted genomic diversity. Here we describe and compare the genome sequences of seven *P. freudenreichii* phages isolated from Swiss-type cheeses and a related prophage in *P. freudenreichii* CIRmBIA139.

## Methods

### Bacterial culture

Bacterial strains (Additional file 1: Table S1) were from either the American Type Culture Collection (ATCC) or from the Collection du Laboratoire de Recherche de Technologie Laitière, Institut National de la Recherche Agronomique, Rennes, France. Colonies were grown on Brucella agar with 5% sheep’s blood, hemin, and vitamin K (Thermo Fisher Scientific, Remel Products, Lenexa, KS, USA) at 30 °C for 5-7 days under anaerobic conditions in a sealed anaerobic box with oxygen-absorbing, carbon dioxide-generating AnaeroPack-Anaero® sachets (Mitsubishi Gas Chemical Co., Inc. [MGC], Tokyo, Japan). Liquid cultures inoculated from single colonies were grown anaerobically at 30 °C to mid-log phase (OD_600_ = 0.4-0.7) in yeast extract sodium lactate (YEL) media, containing (per Liter), 10 g pancreatic digest of casein, 10 g yeast extract, 10 g sodium lactate, 2.5 g KH_2_PO_4_, 5 mg MnSO_4_ [20].

### Isolation of bacteriophage from cheese

The *P. freudenreichii* phages, B22, E1, E6, G4, and B3 were previously isolated from Swiss-type cheese as reported [15, 16]. Two new phages, Anatole and Doucette [21], were isolated at UCLA using a similar protocol. Ten grams of each cheese sample was shredded and dispersed with a Cuisinart Mini-Prep® Plus Processor in 90 ml of YEL media for 5 min. The suspension of cheese + YEL was enriched for 5 days at 30 °C in a sealed box with an AnaeroPack-Anaero® sachet (MGC). The suspension was then centrifuged at 3000Xg for 20 min and filtered through a Corning® 0.2 μm syringe filter (Corning Inc., Corning, NY, USA).

Mid-log phase *P. freudenreichii* TL110 (200 μl at OD_600_ = 0.4-0.7) was incubated with 100 μl filtered supernatant from cheese enrichments for 20 min at 30 °C. This was then plated as a top agar overly in 3 ml molten YEL top agar (0.7% agar) on YEL agar plates (1.5% agar). Plates were incubated for 5 days at 30 °C in a sealed box with an AnaeroPack-Anaero® (MGC). Plates were observed each subsequent day after plating, and plaques were observed on day 5.

Isolated plaques were picked from top agar lawns using a sterile micropipette tip into 100 μl YEL media and stored at 4 °C. These were serially diluted, and 10 μl of each dilution was incubated with 200 μl of TL110 strain at 30 °C for 20 min, and plated using the soft agar method, described above. In total, three rounds of plaque purification were performed for each phage. Lysates were then collected from plates showing a webbed lysis pattern indicative of multiple rounds of phage infection and filtered through a 0.2-μm syringe filter (Corning, Inc.).

### Phage electron microscopy

High titer lysates at 10^9^-10^11^ plaque-forming units (PFU)/ml were subjected to ultracentrifugation at 20,000Xg for 2 h, and the resulting pellets were resuspended in 1 ml phage buffer (10 mM Tris-HCl, pH 7.5; 10 mM MgSO_4_; 68.5 mM NaCl; 1 mM CaCl_2_). Purified phage lysates were spotted in 5 μl aliquots onto freshly glow-discharged 400-mesh carbon-Formvar-coated copper grids and allowed to sit for approximately 1 min. Grids were washed with distilled water and stained using 1% (*w*/*v*) uranyl acetate. Images were captured using an FEI Morgagni transmission electron microscope at 56,000X magnification.

### Phage genome sequencing and analysis

High titer lysates (10^9^-10^11^ PFU/ml) were used for phage DNA extraction. Briefly, DNase I (1 μg/ml final concentration) and RNase I (1 μg/ml final concentration) were added to the lysates for 30 min at room temperature to degrade any host nucleic acids in the lysate. Phage DNA was then isolated using the Promega Wizard DNA Clean Up Kit (Promega Corp., Madison, WI, USA), as described previously [22]. Purified genomic DNA was subjected to whole-genome sequencing using the Illumina MiSeq platform by the Technology Center for Genomics and Bioinformatics (TCGB) at UCLA. The approximate coverage obtained for each phage is as follows: Anatole, 11,161; B3, 9371; B22, 9019; Doucette, 3409; E1, 1000; E6, 14,437; and G4, 13,283. Raw Sequence reads were assembled using Newbler Assembler and Consed or the ABySS software package with manual correction. Genome ends were deduced from large read-start buildups located on opposite strands, separated by a 12-base region of lower coverage. Finished sequences were analyzed and annotated using the genome editor DNAMaster (http://cobamide2.bio.pitt.edu). GenBank accession numbers are listed in Table 1. Genome comparisons were performed and represented using Phamerator and the database ‘Actinobacteriophage_685’ [23].

**Table 1:** Features of newly sequenced *P. freudenreichii* phages

Phage	Source	Year of Isolation	Sensitive strain	Length (bp)	GC%	No, of ORFs	Accession Number
B22	Emmental, French	1992	TL110	37,219	65.45	61	KX620750
Anatole	Emmental, French	2015	TL110	35,284	64.43	55	KX620748
E1	Emmental, French	1992	TL110	35,209	64.42	55	KX620752
Doucette	Emmental, French	2015	TL110	37,403	65.60	60	KX620751
E6	Emmental, French	1992	LS2500/TL110	38,067	65.20	60	KX620753
G4	Emmental, French	1992	Z435/TL19	38,555	65.70	69	KX620754
B3	Comte, French	1992	TL110	35,948	64.40	57	KX620749

## Results

### Isolation of bacteriophages

To investigate the diversity of bacteriophages that infect *P. freudenreichii*, we isolated *P. freudenreichii* phages from Swiss-type cheese by culturing with, or without, indicator strains of host bacteria (Table 1). A total of seven independent bacteriophages isolated from different cheese samples were included in our analyses (Table 1). Phages B22, E1, E6, and G4 were isolated previously from French Emmental-type cheese in 1992, whereas Phage B3 was isolated from French Comté-type cheese in 1991 [15, 16]. These phages, with the exception of G4, were regrown in cultures containing *P. freudenreichii* strain TL110 as the host; phage G4 was grown with TL19 as the host. Phages E6 and G4 were originally propagated on strains, LS2500 and Z435, respectively. The phages named ‘Anatole’ and ‘Doucette’ were isolated in 2015 by processing Emmental-type cheese of French factory origin in YEL media. Enriched cheese filtrates were amplified by infecting a sensitive *P. freudenreichii* strain (TL110, Table 1), and two rounds of phage purification were performed prior to generation of high titer lysates.

### Morphology of *P. freudenreichii* plaques and phage virions

**Fig. 1:**
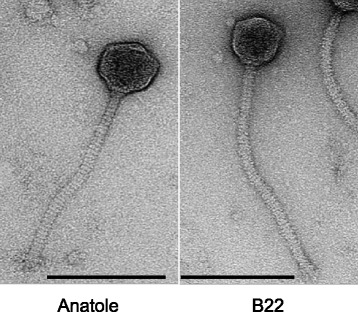
Morphology of *P. freudenreichii* bacteriophages. Electron micrographs of Anatole and B22 (as indicated) showing both have siphoviral morphologies with isometric heads and long flexible tails. Scale markers are 100 nm

Isolated phages infecting *P. freudenreichii* strains (Table 1) were plated onto YEL agar plates, with plaques appearing after culturing for 5 days at 30 °C under anaerobic conditions. All plaques appear relatively clear and small, with similar morphologies and sizes. *P. freudenreichii* strain TL110 was used as the host to prepare high-titer phage lysates with titers 10^9^-10^11^ PFU/ml. These were analyzed by electron microscopy, and all phages were found to have a siphoviral morphology with isometric heads of ~ 50 nm in diameter and long flexible tails (Fig. 1); the tails of Anatole and B22 are ~ 160 nm and ~ 190 nm in length, respectively (Fig. 1). The others are predicted to have similarly sized tails, based on the length of their tape measure genes, the product of which determines phage tail length [24]. The predicted tape measure protein lengths are as follows: 1132 amino acids (aa) in Anatole, E1, and B3; 1326 aa in B22; and 1364 aa in Doucette, G4, and E6. Morphologically, the *P. freudenreichii* phages resemble *C. acnes* phages, which have a slightly shorter tail length of ~ 150 nm [5].

### Host range analysis

To determine the host range specificities of these *P. freudenreichii* phage isolates, we infected different *P. freudenreichii* strains, including TL110, TL18, TL19, and TL29, with phages E6, B3, E1, G4, B22, Doucette, and Anatole. As shown in Table 2, TL18 is resistant to all seven phage isolates, whereas TL110 and TL29 are sensitive to all phages except G4. Of note, G4 is the only phage isolate that can specifically infect TL19, indicating that this phage has a host range that is more selective, and distinct from, than the other *P. freudenreichii* phage isolates. In addition, we also included seven *P. freudenreichii* strains from ATCC in the host range analysis, including ATCC 39393, 9616, 9615, 9614, and 13,273 (subsp. *shermanii*) and ATCC 9617 and 6207 (subsp. *freudenreichii*). Our results indicate that these strains are resistant to infection by all phages tested. Collectively, these host range data demonstrate that the *P. freudenreichii* phages show a higher degree of host specificity than the phages infecting *C. acnes* [5].

**Table 2:** Host preferences of *P. freudenreichii* bacteriophages

	Phage						
Host	Anatole	Doucette	B22	B3	E1	E6	G4
TL110	S	S	S	S	S	S	R
TL29	S	S	S	S	S	S	R
TL18	R	R	R	R	R	R	R
TL19	R	R	R	R	R	R	S
6207	R	R	R	R	R	R	R
9614	R	R	R	R	R	R	R
9615	R	R	R	R	R	R	R
9616	R	R	R	R	R	R	R
9617	R	R	R	R	R	R	R
13,273	R	R	R	R	R	R	R
39,393	R	R	R	R	R	R	R

*S sensitive, *R* resistant

### Genomic DNA characterization of *P. freudenreichii* phages

Total genomic DNA was prepared from high titer *P. freudenreichii* phage lysates, and the complete genome sequences were determined. We observed that the genome sizes among these sequenced phages are similar and vary from 35,209 bp to 38,555 bp, which is larger than those of the *C. acnes* phages that average 29,432 bp in length. However, the overall genome sizes for the *P. freudenreichii* phages and *C. acnes* phages are only about half the average size of the mycobacteriophages, whose hosts are related members of the high GC Gram-positive Actinobacteria [25]. All *P. freudenreichii* viral genomes have defined ends with 12 bp 3′ single-stranded extensions, although the sequences vary among the phages: 5’-CAAACAAGTCAT in Doucette, 5’-CCAACAAGTCAT in B22, E6 and G4, and 5’-CTCCCGCTCGAA in Anatole, B3, and E1.

The percent GC content was also observed to vary little among the *P. freudenreichii* phages, ranging from 64.40% to 65.70% GC. The average percent GC content of *P. freudenreichii* phages is 64.94%, similar to that of their *P. freudenreichii* bacterial hosts (67.3%) and other *Propionibacterium* species, which range from 53%-68% [26]. This is in contrast to the *C. acnes* phages, which show a more restricted range of GC% content (53.76%-54.37%) with an average of 54.04%, considerably lower than the ~ 60% GC content of its host *C. acnes* [5]. The low variation in the GC% contents of *P. freudenreichii* phages suggests that they may have shared phylogenetically similar hosts in their recent evolutionary histories [27].

### Nucleotide sequence identity among *P. freudenreichii* phages

**Fig. 2:**
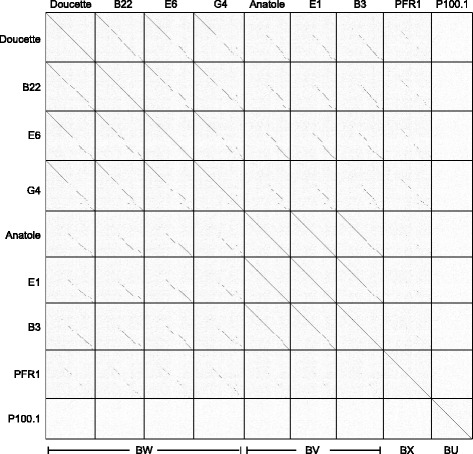
Dot plot nucleotide comparisons of *P. freudenreichii* bacteriophages. The genome sequences of seven *P. freudenreichii* bacteriophages (varying from 35,209 bp to 38,555 bp) were compared with each other, as well as the previously sequenced *P. freudenreichii* phage, PFR1 (38,071 bp) [19] and the *C. acnes* phage, P100.1 (29,612 bp) [5], using the dotplot program Gepard, with standard parameter settings (word length = 10, window = 0) [39]. Cluster BW includes Doucette, B22, E6, and G4. Cluster BV includes Anatole, E1, and B3. PFR1 and P100.1 group in the separate clusters, BX and BU, respectively

Fully sequenced genomes for the seven *P. freudenreichii* phages sequenced in this study, as well as the previously sequenced *P. freudenreichii* phage, PFR1 [19], and the *C. acnes* phage, P100.1 [5], were compared according to their shared genome contents and average nucleotide sequence similarities (Fig. 2, Additional file 1: Table S2). The phages all share some nucleotide sequence similarity, although there is more extensive similarity among phages Doucette, B22, E6, and G4, compared to Anatole, E1, and B3, which also share extensive similarity with each other. For example, Doucette and B22 share 97% identity, with a coverage of 76%; whereas Doucette and Anatole share 91% identity, but with a coverage of only 24%. Conversely, Anatole and B3 share 98% identity with a coverage of 91%. On this basis we propose organizing the phages into two clusters based on previously described criteria, with span-length match being the primary factor that distinguishes the two clusters (as described further below) and cluster names deriving from the Actinophage Database [28, 29]: Cluster BW contains Doucette, B22, E6, and G4, and Cluster BV contains Anatole, E1, and B3 (Fig. 2). We further note that the previously characterized *P. freudenreichii* phage, PFR1, is more distantly related to the phages sequenced in this study and groups in a separate cluster designated BX. From the dotplot, we can observe discrete regions of nucleotide identity between PFR1 and the Cluster BW phages (Doucette, B22, E6, and G4) and more limited identity between PFR1 and the Cluster BV phages. No observable identity similarity can be detected between *C. acnes* phage P100.1 and the group of *P. freudenreichii* phages.

**Fig. 3:**
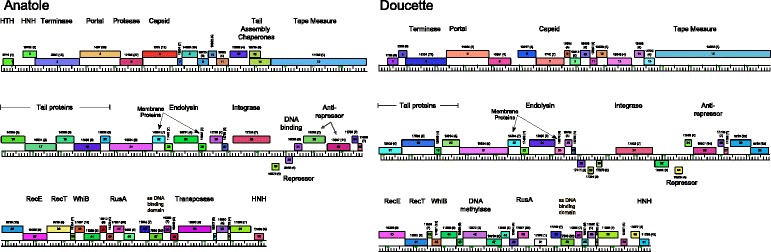
Genome map of *P. freudenreichii* phages Anatole and Doucette. Predicted genes are shown as boxes either above or below the genome corresponding to rightwards- and leftwards-transcription, respectively. The gene numbers are shown within each colored box, and its phamily number is shown above, with the number of phamily members shown in parentheses; coloring reflects the phamily assignment. Grouping of genes into phamily of related sequences and map generation was performed using Phamerator [40] and the database ‘Actinobacteriophage_685’. Putative gene functions are listed above the genes

The members of Cluster BW share ~ 90% average nucleotide identity (Additional file 1: Table S2), but as illustrated by comparison of B22 and Doucette, there are regions of near-identity interspersed with regions that are more diverse (Fig. 2). The left end of E6 is very similar to that of Doucette, but the right ends are more diverse (Fig. 2). The Cluster BV genomes share > 96.4% average nucleotide identity to one another (Additional file 1: Table S2). Of note, phages Anatole and E1 have ~ 99% identity at the nucleotide level and differ by only two single nucleotides, despite the fact that E1 was isolated in France in 1995 [16] and Anatole was isolated in the United States in 2014. B3 has 96.5% identity to E1 in overall genome sequence, although there are several gaps or regions of difference between the two genomes, particularly in the right genome arms, as can be seen in the dotplot in Fig. 2. B3 is also slightly longer than E1, with genome lengths of 35,948 bp and 35,209 bp, respectively. The nucleotide sequence identities between Clusters BW and Cluster BV range from 82% to 96%, but the matching regions only span 31-45% of the genome lengths (aligned using BLASTN), mostly in the 3′ halves of the genomes (Fig. 2). These parameters justify the separation of the phages into the separate BW and BV clusters. The annotated genomes of Anatole and Doucette are shown in Fig. 3, and genome annotations of B22, E6, G4, E1, and B3 are shown in Additional file 2: Figure S1, Additional file 3: Figure S2, Additional file 4: Figure S3, Additional file 5: Figure S4, and Additional file 6: Figure S5, respectively.

### Genome annotations of Cluster BW *P. freudenreichii* phages

**Fig. 4:**
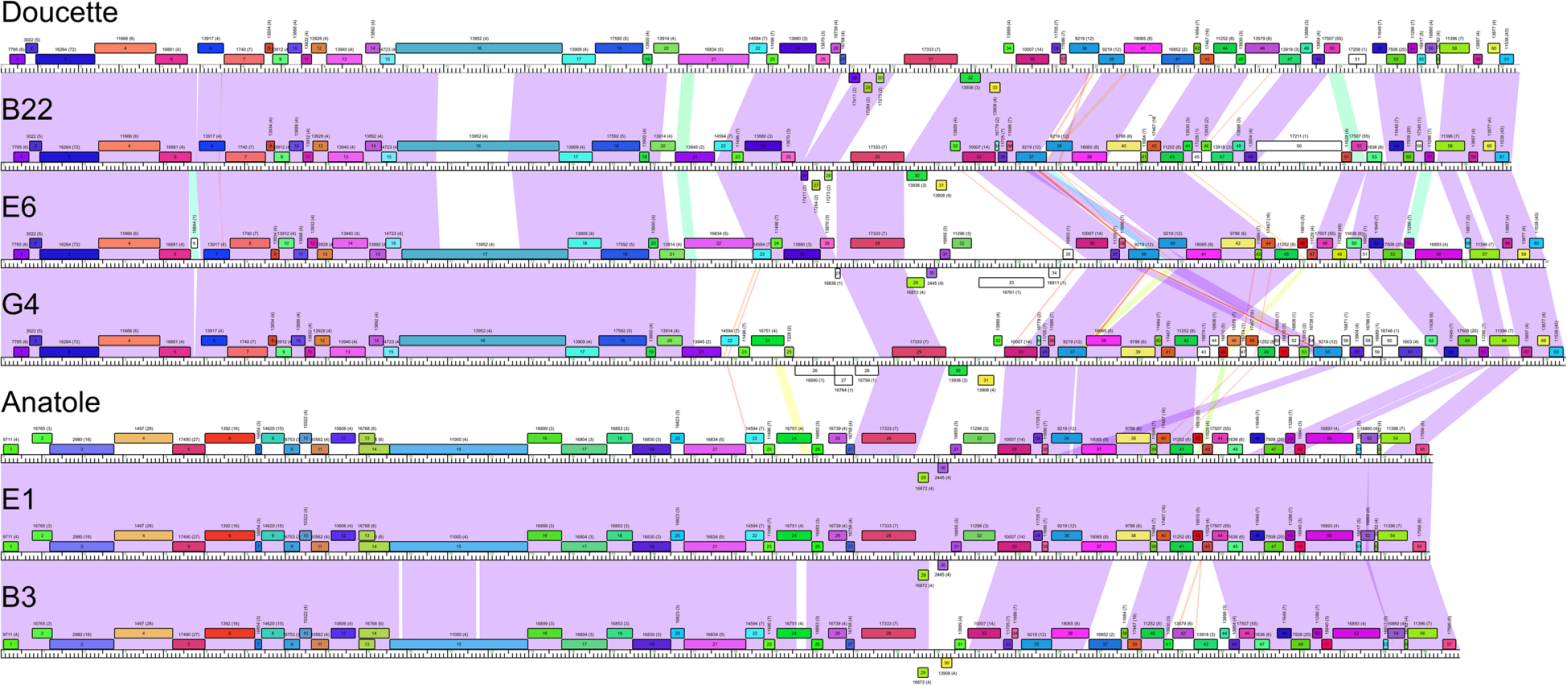
Whole genome comparisons of *P. freudenreichii* bacteriophages. The genome organizations of seven *P. freudenreichii* bacteriophages are shown with the pairwise nucleotide sequence similarities displayed as colored segments between the genomes. The strength of sequence similarity is represented according to a color spectrum in which violet is the most similar and red is the least (above a threshold BLASTN E-value of 10^− 5^). Predicted genes are shown as colored boxes either above or below each genome, representing leftwards- and rightwards-transcription, respectively. The gene numbers are shown within each colored box, and its phamily number is shown above, with the number of phamily members shown in parentheses; coloring reflects the phamily assignment. The maps were generated using the program Phamerator [40] and database Actinobacteriophage_685

Annotation of the open reading frames in the Doucette genome, a 37,429 bp representative from Cluster BW, reveals 61 predicted protein-coding genes (Fig. 3, Table 1). The overall genome organizations of Doucette and other members of Cluster BW (B22, E6, and G4; Additional file 2: Figure S1, Additional file 3: Figure S2, Additional file 4: Figure S3) are similar to each other, although there are notable differences, particularly in the right arms of the genomes (Fig. 4). The virion structure and assembly genes are organized in the left arms of the genomes. We note that the portal and major capsid proteins are related to those in mycobacteriophage Gaia (62% and 37% aa identity, respectively), and the major tail subunits have C-terminal Ig-like domains, as described for other phage structural proteins [30]. The Doucette and E6 tail proteins (gp21 and gp22, respectively) differ from B22 and E6 (gp21) in that they contain a glycosyl hydrolase family 43 domain that is typically an endolysin component. These are likely to be tail proteins, however, as the lysis cassette is readily identifiable and is located further to the right in the genomes. Although this glycosyl hydrolase domain is atypical of phage tail proteins, we note that several mycobacteriophage tail proteins contain putative D-ala-D-ala carboxypeptidase domains [31]. We therefore predict that the cell wall target of these enzyme activities may be cleaved or remodeled during the process of phage adsorption and DNA injection. The lysis cassette also varies among the Cluster BV phages, with G4 having a different endolysin and holin gene than is found in Doucette, B22, and E6 (Fig. 3, Additional file 2: Figure S1, Additional file 3: Figure S2, Additional file 4: Figure S3).

The Cluster BW phages have several genes near the center of the genomes that are typically associated with temperate growth (e.g., putative integrase and repressor). Doucette gp31 is a tyrosine integrase, which is more distantly related to the integrases encoded by the other Cluster BW and BV phages than these proteins are to one another. That is, the predicted integrase proteins from B22 (gp29), E6 (gp28), and G4 (gp29), as well as those from Anatole (gp28), E1 (gp28), and B3 (gp28) share ~ 96-99% aa identity, whereas they have only ~ 54-55% sequence identity to Doucette gp31. Comparison of the sequences upstream of Doucette gene *31* with *P. freudenreichii* genomes identified a 45 bp region of sequence identity corresponding to the common core *attP* (Doucette coordinates 22,180 – 22,224) and an *attB* site (coordinates 934,527 – 934,571 in *P. freudenreichii* subsp. shermanii CIRM-BIA1) overlapping a tRNA^pro^ gene. We predict that Doucette integrates at this *attB* site in lysogenic establishment. The other Cluster BW genomes differ in their sequences upstream of the integrase gene, and a similar analysis identified a 49 bp common core corresponding to the *attP* common core in B22 (coordinates 20,750 – 20,798) and an *attB* in the *P. freudenreichii* genome (subsp. shermanii CIRM-BIA1, coordinates 376,562 – 376,610), such that the *attB* site overlaps a tRNA^gly^ gene. The three Cluster BV phages have closely related *attP* sites and integrase genes and are predicted to integrate at the same *attB* site.

Three of the Cluster BW phages (Doucette, B22, and G4) encode putative repressors (genes *33*, *31*, and *31*, respectively) that are identical to each other, and also identical to the repressor (gp30) of Cluster BV phage B3. We therefore predict that these form a homoimmune group of temperate phages. The E6 repressor (gp30) differs from these (40% aa identity), but is identical to the repressors of Cluster BV phages Anatole and E1, likely forming a separate homoimmune group. In each of the genomes, the repressor is transcribed leftwards and divergently from an operon of rightwards-transcribed genes, in which the first is a putative *cro*-like gene. There is considerable variation in the Cluster BW gene content in the Integrase/Repressor regions. G4 contains three leftwards-transcribed genes (*26*, *27*, *28*) of unknown function (Additional file 4: Figure S3) to the left of the integrase, and there are three small genes common to Doucette (*28*, *29*, *30*) and B22 (*26*, *27*, *28*) in this region that are absent from the other phages; the function of these is also unknown. E6 is unusual in that it contains a second set of divergently transcribed genes (*34* and *35*) organized as an additional repressor-*cro* pair of genes, suggesting that it may have complex immunity properties. It also has an insertion of a large gene (*33*) that contains a putative DNA binding motif but is otherwise of unknown function.

The Cluster BW right arm genes also vary among the genomes, although they all code for a RecET-like system, a WhiB-like regulator, a single-stranded DNA binding protein, and an HNH endonuclease (Fig. 3, Additional file 2: Figure S1, Additional file 3: Figure S2, Additional file 4: Figure S3); E6 contains a transposase gene (gp55) of the IS*1341* group of insertion sequences [32] (Additional file 3: Figure S2). Doucette and B22 both encode DNA methylase genes, although they are not related to each other and are at different genomic loci. We note that, just downstream of the putative antirepressor genes (e.g. Doucette *35*), Doucette, B22, and E6 contain two genes that are tandemly repeated (e.g. Doucette *38* and *39,* B22 *37* and *38,* and E6 *39* and *40*); this gene is also duplicated in G4, but the two copies, genes *37* and *55*, are separated by ~ 5.5 kb. However, the intergenomic similarities are closer than the intragenic similarities (e.g. Doucette gp38 and gp39 share 55% aa identity, and Doucette gp38 and B22 gp39 share 93% identity; Additional file 7: Figure S6).

### Genome annotations of Cluster BV *P. freudenreichii* phages

Annotation of the Anatole genome, a 35,284 bp representative of Cluster BV, reveals 55 predicted protein-coding genes, and no tRNA genes (Fig. 3, Table 1). The genome organizations of Anatole (Fig. 3) and E1 (Additional file 5: Figure S4) are near-identical in their annotations, but differ from B3 (Additional file 6: Figure S5) at several locations. The virion structure and assembly genes are organized in the genome left arms (Anatole genes *3* – *21*) and arranged syntenically with structural genes of other phages with siphoviral morphologies. Several of the structural proteins (e.g. portal, prohead protease, capsid, major tail subunit) have sequence similarity to phages of other actinobacterial hosts, including Cluster N mycobacteriophages, corynebacteriophage BFK20, and microbacteriophage Min1, although amino acid sequence similarities are modest (27-45% aa identity). The minor tail proteins implicated in host recognition are not related to other phages other than Anatole gp21, which is related to the tail proteins of Cluster BW phages Doucette and E6. The lysis cassette – including endolysin and holin genes (Anatole *24* and *25*, respectively) – are located to the right of the virion structural genes (Fig. 3).

Similar to the Cluster BW phages, the centers of the genomes include several genes associated with temperate growth. There are integration cassettes containing a tyrosine-integrase, with an *attP* located upstream, as described for Cluster BW phages B22, E6, and G4. The Anatole 49 bp *attP* common core is located at coordinates 21,035 – 21,083. To the right of the integration genes are two leftwards-transcribed genes, including the likely phage repressor (Anatole *30*), which is divergently transcribed from a *cro*-like gene (*31*); immediately downstream are two genes (*32*, *33*) related to anti-repressors (Fig. 3). As noted above, the Anatole and E1 repressors are identical to those of BW phage E6, whereas the B3 repressor is identical to the Doucette, B22, and G4 repressors. Phage B3 differs from Anatole and E1 in that it contains only a single antirepressor gene (Additional file 6: Figure S5).

The genes in the rightwards-transcribed right arm include a RecET-like recombination system (Anatole gp37, gp38), a Holliday Junction resolvase (Anatole gp44), a WhiB-like regulator (Anatole gp40), a single stranded DNA binding protein (Anatole gp47), and a transposase (Anatole gp50) of the IS*1341* group of insertion sequences [32]. As noted above, this transposase is also found in E6 (gp55). It is currently unclear whether this transposon may be active, as we have not been able to identify flanking inverted repeats flanking the transposon in any phage in which it is located. Phage B3 differs from Anatole and E1 in the right arm in two aspects. First, it contains a different *recT*-like gene (B3 gene *37*; Additional file 6: Figure S5) that is more similar to Doucette *41* (63% aa identity) than to the Anatole and E1 *recT* genes. Second, genes *42* and *43* of Anatole and E1 are replaced with B3 genes *41* – *45*, all of which have closely-related homologues in Doucette and four genes (with the exception of B3 *42*) in B22 (76 – 100% aa identity). Most are of unknown function, although G3 gp42 is a probably a DNA N6-methyltransferase.

### Comparison between *P. freudenreichii* phages and other phages

**Fig. 5:**
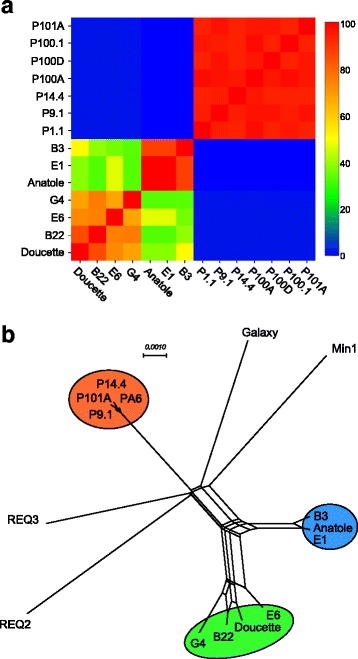
Relationships between Propionibacteriophages and Cutibacteriophages. **a** Heat map representation of shared gene content between seven *P. freudenreichii* phage genomes and seven *C. acnes* phage genomes. The proteins encoded by *C. acnes* phages (P101A, P100.1, P100D, P100A, P14.4, P1.1) and *P. freudenreichii* phages (Cluster BW: G4, E6, B22, Doucette; Cluster BV: B3, E1, Anatole) were assorted into phamilies using Phamerator [40] and the proportions of shared genes calculated. The scale on the right shows heat map colors with percent of shared genes. **b** Gene content relationships between *P. freudenreichii* phages and phages of related hosts. The relationships between the seven *P. freudenreichii* phages and a selection of phages that infect other bacteria in the Actinobacteria phylum were compared according to shared gene content using Phamerator and displayed using the Neighbor Network function of SplitsTree [34]. Cluster BW phages of *P. freudenreichii* are shown in the green circle, Cluster BV phage of *P. freudenreichii* in the blue circle, and *C. acnes* phages in the orange circle. Galaxy and Min1 are phages of *Mycobacterium* and *Microbacterium,* respectively, and REQ2 and REQ3 are *Rhodococcus* phages

*P. freudenreichii* phages were also compared with other phages in terms of their genome structures and sequences, especially those that infect the related host, *C. acnes*. Although these two groups of phages are not closely related at the sequence level, the overall arrangement of genes involved in packaging, head assembly, tail assembly, lysis, regulation, and replication is similar between *P. freudenreichii* phages and *C. acnes* phages; the *C. acnes* phages, however, lack genes for lysogenic growth. While most of the *P. freudenreichii* phage genes are transcribed in the rightwards direction, the non-structural genes are transcribed leftwards in the *C. acnes* phages [5]. We noted previously that *C. acnes* phages display only very limited diversity, which is in marked contrast to the *P. freudenreichii* phages that not only show a greater diversity, but also considerable genome modularity, consistent with the mosaic architectures observed in other phage populations [33]. In particular, there has been considerable swapping of genes between the Cluster BW and BV phages, illustrated notably by the distribution of immunity and integrase functions. This difference in degrees of diversity is evident in a heat map showing pairwise shared gene content between the *C. acnes* and *P. freudenreichii* phages (Fig. 5a). The phylogenetic relationship between the phages (Fig. 5b) is illustrated by a network analysis using SplitsTree [34], where the Cluster BV and Cluster BW phages are on distinct branches, and both are distantly related to other phages of the Actinobacteria phylum (Fig. 5b).

### Similarities to *P. freudenreichii* prophages

**Fig. 6:**
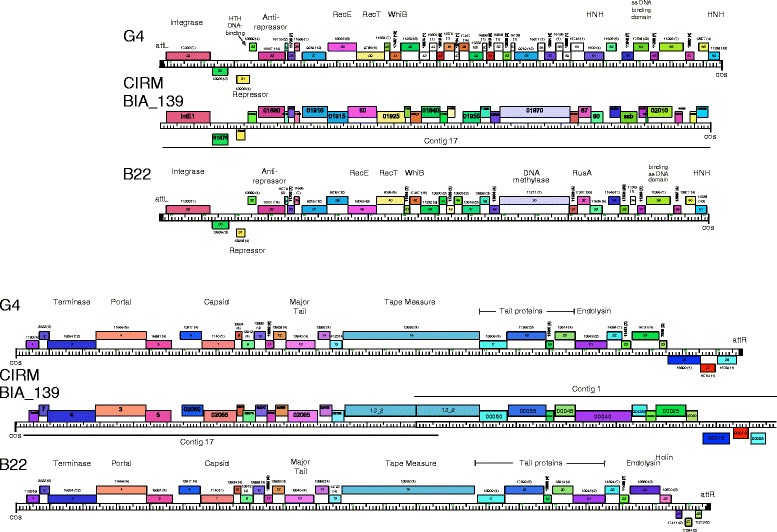
The mosaic prophage of *P. freudenreichii* CIRM-BIA 139. The whole genome sequence project of *P. freudenreichii* contains two contigs (contigs 1 and 17; Accession numbers CCYX01000001 and CCYX01000017, respectively) with similarities to Cluster BW phages. The predicted organization of the prophage is shown aligned to phages G4 and B22 presenting them in two tiers, and organization from *attL* through to *attR* reflecting the integrated prophage structure. Segments corresponding to the two contigs are shown, which overlap in the middle of the tape measure protein gene; the reverse complement of contig 1 is shown. The prophage genes are colored according to their homologues in G4 and B22. The genes in prophage contig 1 are either shared with both phages or only with phage B22, whereas the genes at the right end of contig 1 are shared uniquely with phage G4

Comparison of *the P. freudenreichii* phage genome sequences reveals several close matches to *P. freudenreichii* genomes corresponding to integrated prophages. The *P. freudenreichii* CIRM-BIA 139 genome includes two regions (contigs 1 and 17) matching the Cluster BW phages that together span the entire prophage from the *attL* left integration site to the rightmost integration site, *attR* (Fig. 6). Interestingly, the prophage is most closely related to the Cluster BW phages, but is mosaic, and contains parts of the individual phages but in a new combination. The prophage segment from *attL* to *cos* is most similar to B22, and most of the predicted gene products have amino acid sequences with close to 100% identity, including the DNA methylase. The putative repressor is 100% identical to those predicted in G4 and B22, and thus, the prophage likely shares immunity with the BW phages. The region from *cos* to the tape measure protein gene is similar in all four Cluster BW phages, but the genes from the tape measure protein gene to *attR* have more complex relationships, with CIRM-BIA 139 contig 1 *00035* and *00030* gene products being 100% identical to B22 gp22 and gp23, respectively (Fig. 6), whereas the prophage genes *00025* - *00005* are similar to genes *24*-*28* of phage G4 (89 – 97% amino acid identity). *P. freudenreichii* strain CIRM-BIA 119 appears to carry an identical prophage to the one in BIA 139, and also spans two contigs. Searching with the Anatole genome did not identify any additional prophages.

## Discussion

The Propionibacteriaceae family is comprised of a diverse group of organisms, containing species such as *C. acnes,* a human commensal proposed to be involved in the pathogenesis of acne vulgaris, and *P. freudenreichii,* which is used in the manufacture of Swiss-type cheeses, as well as in the fermentative production of vitamin B_12_ and propionic acid. In both cases, the roles of phages targeting these bacterial hosts have not been well defined. For example, it is unclear how the resident *C. acnes* phage population shapes that of its host to promote health or disease. *P. freudenreichii* phages are often present during cheese ripening and can be isolated from mature cheeses; the semi-solid state of the cheese paste likely acts to limit phage diffusion, allowing phages to co-exist within large populations of sensitive hosts [16]. Recent phage genome sequencing efforts by us and others have revealed a strikingly limited diversity amongst *C. acnes* phages, and phenotypic analyses indicate that these phages generally display broad host ranges, with most *C. acnes* strains being highly phage sensitive [5, 35, 36]. In contrast, despite the report of a single genome sequence for a ssDNA filamentous phage active on *P. freudenreichii* [17] and the recently published genomes of two highly similar dsDNA *P. freudenreichii* phages [19], little is known about the genetic composition of the phages infecting this organism and whether they share features in common with *C. acnes* phages. Although these two bacterial species have recently been re-classified into distinct genera [4], their shared metabolic features (e.g. generation of propionic acid) and degree of evolutionary relatedness, support the potential utility of comparing their respective phages.

Here, we report the full-length genome sequences for seven dsDNA tailed *P. freudenreichii* phage isolates. Sequence analysis indicates that these phages fall into two major clusters with more divergent nucleotide and amino acid sequences, as compared to the *C. acnes* phages. Further, *P. freudenreichii* phages display more restricted host ranges, with most *P. freudenreichii* strains showing high levels of phage resistance. Notably, although we obtained over 80 cheese samples from across the United States, Switzerland, and France, which were originally produced in the United States, France, Switzerland, Spain, and Italy, we were only able to isolate phages from Swiss-type cheeses produced in French factories. The reason for this is unclear, although some of this difficulty is likely due to low levels of phage contamination within individual samples, as well as the lack of phage sensitivity displayed by most *P. freudenreichii* strains. Because the ‘eyes’, or ‘holes’ in Swiss-type cheese arise due to the CO_2_ byproduct produced from the added *P. freudenreichii* strains, cheeses with fewer to no holes present might indicate higher likelihood of phage contamination and/or the use of bacterial strains that are more phage sensitive. Few studies have investigated the microbial composition of cheese, although, a recent analysis of the cheese-rind microbiome from over 130 samples of diverse origin has revealed group of 24 widely distributed bacterial and fungal genera [37]. This group does not include *Propionibacterium,* although members of this genus were detected at low abundance in numerous samples. We therefore searched our phage genome sequences against a subset of the cheese-rind datasets, focusing on those from French cheeses, those with a natural rind (as is present on Swiss-type cheeses), and samples containing representatives from the *Propionibacterium* genus. From these analyses, we detected a small number of 100 bp reads with > 40% identity to our *P. freudenreichii* phages in five of the samples, however, coverage ranged from 0.3-4.2%, which was not sufficient to map these reads onto any one phage genome.

*P. freudenreichii* phages share limited genetic similarity with phages infecting other members of the Actinobacteria phylum, including the Cluster N mycobacteriophages, the corynebacteriophage BFK20, and microbacteriophage Min1. When the *C. acnes* phages are used as a model for comparison, we observe little to no relationship between these two groups of phages, although they display similar genome architectures. Further, *P. freudenreichii* phages and *C. acnes* phages share virtually no genes in common, and while *C. acnes* phages demonstrate a strikingly limited diversity, *P. freudenreichii* phages contain more diverse genomes. Of particular interest is the observation that whereas all known *C. acnes* phages lack putative lysogeny functions [5, 35, 38, 39], the *P. freudenreichii* phages characterized here all encode putative tyrosine integrases and repressor proteins, strongly suggesting they are temperate.

Our host range analyses also revealed critical differences between these two groups of phages. Whereas *C. acnes* phages generally show broad host ranges, infecting most laboratory and clinical *C. acnes* isolates, the host ranges of *P. freudenreichii* phages are more restricted, and many bacterial strains show high levels of phage resistance. This may result from the presence of prophages in some *P. freudenreichii* strains, which has been suggested experimentally [15, 18] and is further supported by the presence of prophages closely related to the Cluster BW phages in the fully sequenced strains, CIRM-BIA 139 and CIRM-BIA 119. In contrast, and consistent with their broad host ranges, *C. acnes* prophages do not resemble any of the sequenced *C. acnes* phages [5, 35, 38, 39]. We therefore speculate that superinfection immunity due to the presence of prophages in *P. freudenreichii* strains could contribute to the observed host range restrictions, and although we did not detect spontaneous release of viable phages from our host strains (L. Marinelli, data not shown), further research will be needed to rule out this possibility.

Finally, it is also possible that the lack of phage sensitivity among strains of *P. freudenreichii* may also be due, in part, to other factors, such as increased phage receptor diversity on the bacterial cell surface and/or the presence of restriction/modification systems in these bacteria. In addition, a number of *P. freudenreichii* strains are predicted to contain clustered regularly interspaced short palindromic repeat (CRISPR) loci, as predicted by the CRISPRfinder online program (http://crispr.i2bc.paris-saclay.fr/Server/index.php, data not shown), and these may play a role in the defense against phage infection in these bacteria. Thus, future studies will focus on investigating this possibility and determining the mechanism(s) that contribute to phage resistance in *P. freudenreichii*.

Collectively, these findings suggest substantial differences in the evolution of *C. acnes* vs. *P. freudenreichii* and their phages, and we hypothesize that these have arisen due to the distinct ecological niches inhabited by these organisms. *C. acnes* reside within the pilosebaceous follicles of human skin, a lipid-rich, oxygen-limited environment that restricts growth of most other organisms. As such, this bacterium and its phages have likely evolved in isolation with few opportunities for genetic exchange and recombination, which may be reflected in the limited diversity and lack of lysogeny in this population. Conversely, *P. freudenreichii* and its phages are present both in soil environments and in industrial settings, and are also likely to transit through the human GI tract, and in all of these places, they are likely to encounter numerous other microbial species. Thus, we postulate that this dynamic environment has selected for increased diversity and the potential for lysogeny in this group of phages. Future investigations will be focused on determining the frequency with which these phages are able to lysogenize sensitive host strains and whether widespread lysogeny is a primary mediator of the restricted host ranges observed for these phages.

## Conclusions

This report, by describing the complete genomes of seven dsDNA *P. freudenreichii* phages, has broadened our understanding of the phages specific for members of the Propionibacteriaceae family [19]. We propose that these data, together with the existing *P. freudenreichii* phage genomes, may be utilized to further explore how phages influence the production of Swiss-type cheeses and potentially, to develop tools for accelerating *Propionibacterium*-mediated cheese ripening, which is an expensive step during cheese production. In addition, our host range data have identified bacterial strains that are highly resistant to different types *P. freudenreichii* phages. Unlike the *C. acnes* phages, all sequenced *P. freudenreichii* phages contain genes encoding a putative integrase, and our data suggest that lysogeny may be a strategy commonly employed by these phages. Therefore, this information could be utilized to design integration vectors, a system that has been widely employed for genetic manipulation in the related mycobacteria [31], and which may be used to improve the limited genetic tools available for both *P. freudenreichii* and *C. acnes*.

## Additional files

Additional file 1: **Table S1.** *P. freudenreichii* strains used in this study. **Table S2.** Average nucleotide identities of *P. freudenreichii* phages. (DOCX 71 kb)

Additional file 2: **Figure S1.** Genome organization of phage B22. Predicted genes are shown as boxes either above or below the genome corresponding to rightwards- and leftwards-transcription, respectively. The gene numbers are shown within each colored box and its phamily number is shown above with the number of phamily members shown in parentheses; coloring reflects the phamily assignment. Grouping of genes into phamily of related sequences and map generation was performed using Phamerator (30) and the database ‘Actinobacteriophage_685’. Putative gene functions are listed above the genes. (PDF 164 kb)

Additional file 3: **Figure S2.** Genome organization of phage E6. See Additional file 2: Figure S1 for details. (PDF 161 kb)

Additional file 4: **Figure S3.** Genome organization of phage G4. See Additional file 2: Figure S1 for details. (PDF 185 kb)

Additional file 5: **Figure S4.** Genome organization of phage E1. See Additional file 2: Figure S1 for details. (PDF 152 kb)

Additional file 6: **Figure S5.** Genome organization of phage B3. See Additional file 2: Figure S1 for details. (PDF 159 kb)

Additional file 7: **Figure S6.** Phylogenetic tree of tandemly repeated genes in *P. freudenreichii* phages. The four Cluster BW phages all encode two copies of related genes that are tandemly repeated in Doucette, B22, and E6, but which are separated by 17 genes in G4. The Cluster BV phage Anatole contains a single copy of this gene. Genes duplicated in one genome (e.g. Doucette *38* and *39*) are more distantly related (51% aa identity) that with the corresponding gene in another genome (e.g. Doucette gp39 and B22 gp38 share 93% aa identity). Sequences were aligned using ClustalX and the tree drawn using NJPlot. Bootstrap values from 1000 iterations are shown. (PDF 22 kb)

## Abbreviations

ATCC: American type culture collection
CRISPR: Clustered regularly interspaced short palindromic repeat
MGC: Mitsubishi gas chemical co.
PFU: Plaque-forming units
TCGB: Technology center for genomics and bioinformatics
YEL: Yeast extract sodium lactate
