# Supplementary material for: Complete genomic sequences of Propionibacterium freudenreichii phages from Swiss cheese reveal greater diversity than Cutibacterium (formerly Propionibacterium) acnes phages
Source: BMC Microbiol. 2018 Mar 1;18:19. doi: 10.1186/s12866-018-1159-y (PMC5831693; doi:10.1186/s12866-018-1159-y)
Supplement: Supplementary file 1 — Table S1. P. freudenreichii strains used in this study. Table S2. Average nucleotide identities of P. freudenreichii phages. (DOCX 71 kb) [file 12866_2018_1159_MOESM1_ESM.docx]

**Supplementary Information**

**Table S1.** *P. freudenreichii* strains used in this study.

| Strain Name | Subspecies |
| --- | --- |
| TL110 | *shermanii* |
| TL29 | Unknown |
| TL18 | Unknown |
| TL19 | Unknown |
| ATCC 6207 | *freudenreichii* |
| ATCC 9617 | *freudenreichii* |
| ATCC 9614 | *shermanii* |
| ATCC 9615 | *shermanii* |
| ATCC 9616 | *shermanii* |
| ATCC 13673 | *shermanii* |
| ATCC 39393 | *shermanii* |

**Table S2.** Average Nucleotide Identities of *P. freudenreichii* phages.

|  | **Doucette** | **B22** | **E6** | **G4** | **Anatole** | **E1** | **B3** |
| --- | --- | --- | --- | --- | --- | --- | --- |
| **Doucette** | 1 | 0.894 | 0.908 | 0.909 | 0.76 | 0.76 | 0.776 |
| **B22** | 0.894 | 1 | 0.901 | 0.911 | 0.857 | 0.86 | 0.816 |
| **E6** | 0.908 | 0.901 | 1 | 0.905 | 0.834 | 0.836 | 0.782 |
| **G4** | 0.909 | 0.911 | 0.905 | 1 | 0.757 | 0.759 | 0.774 |
| **Anatole** | 0.76 | 0.857 | 0.834 | 0.757 | 1 | 1 | 0.964 |
| **E1** | 0.76 | 0.86 | 0.836 | 0.759 | 1 | 1 | 0.965 |
| **B3** | 0.776 | 0.816 | 0.782 | 0.774 | 0.964 | 0.965 | 1 |
