# Supplementary figures and images for: Complete genomic sequences of Propionibacterium freudenreichii phages from Swiss cheese reveal greater diversity than Cutibacterium (formerly Propionibacterium) acnes phages
Source: BMC Microbiol. 2018 Mar 1;18:19. doi: 10.1186/s12866-018-1159-y (PMC5831693; doi:10.1186/s12866-018-1159-y)

B22

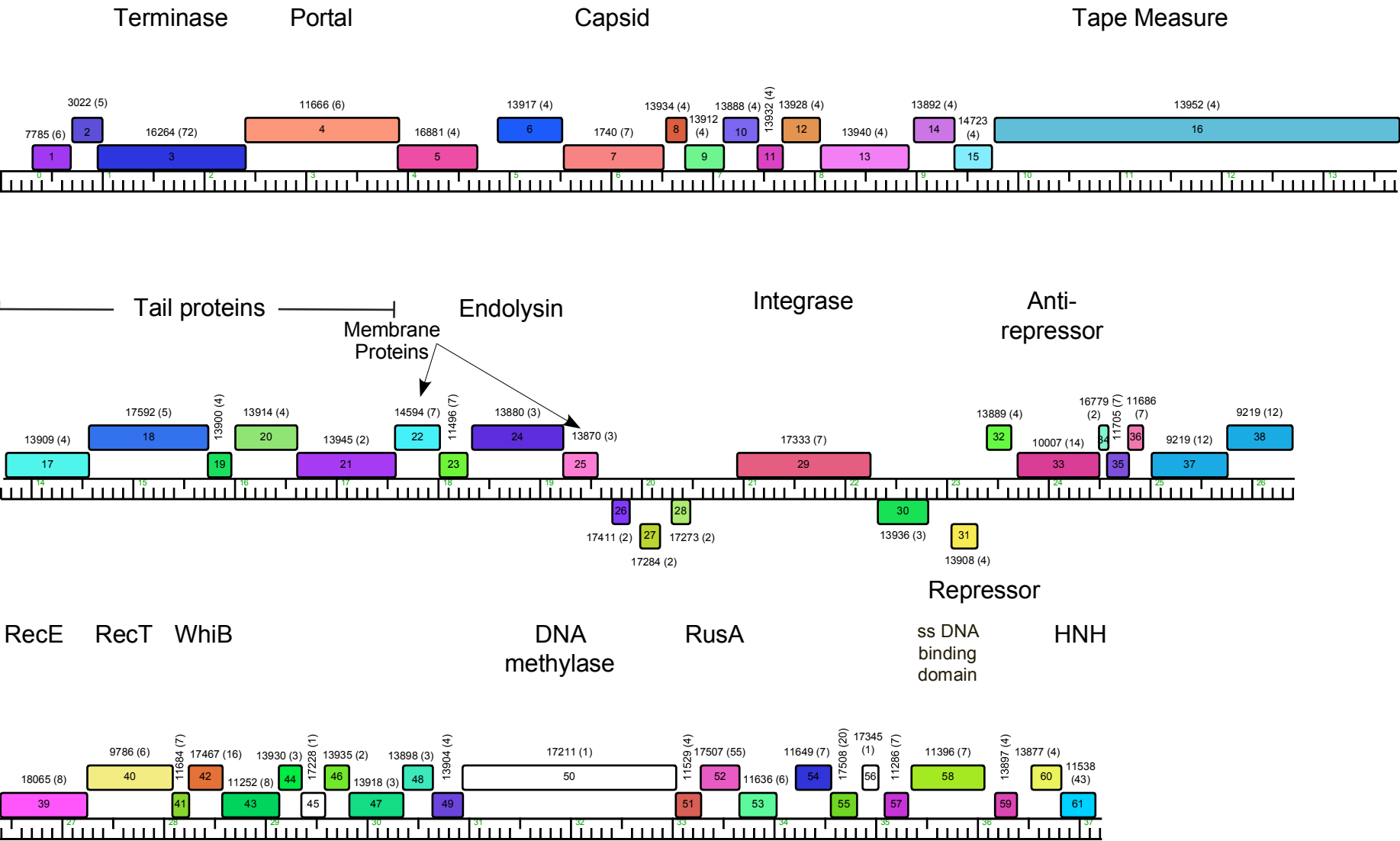

Supplement: Supplementary file 2 — Figure S1. Genome organization of phage B22. Predicted genes are shown as boxes either above or below the genome corresponding to rightwards- and leftwards-transcription, respectively. The gene numbers are shown within each colored box and its phamily number is shown above with the number of phamily members shown in parentheses; coloring reflects the phamily assignment. Grouping of genes into phamily of related sequences and map generation was performed using Phamerator (30) and the database ‘Actinobacteriophage_685’. Putative gene functions are listed above the genes. (PDF 164 kb) [file 12866_2018_1159_MOESM2_ESM.pdf]

# E6

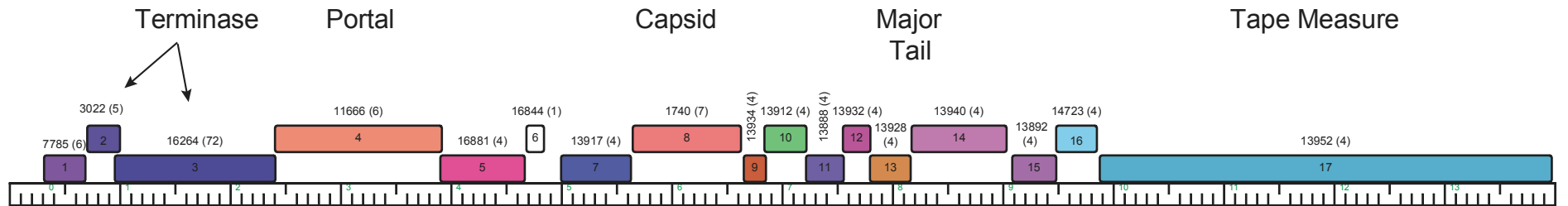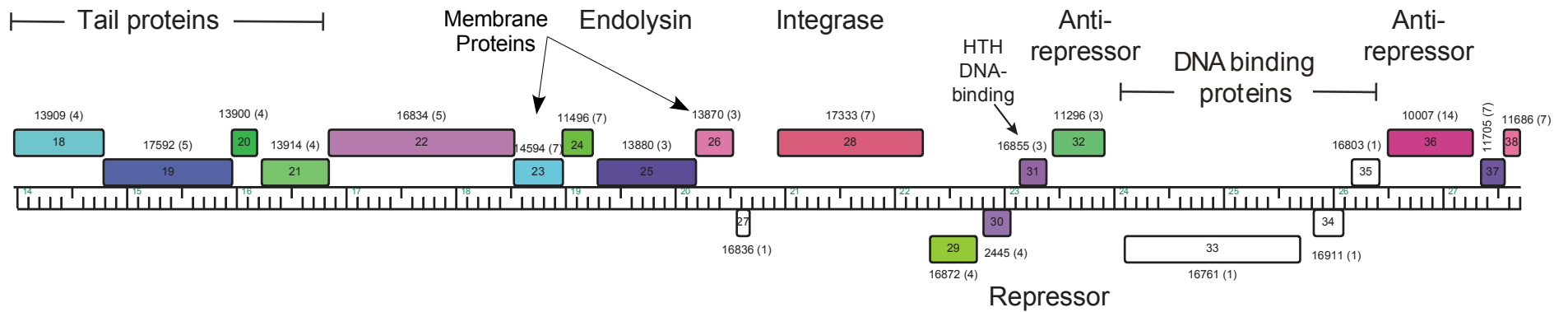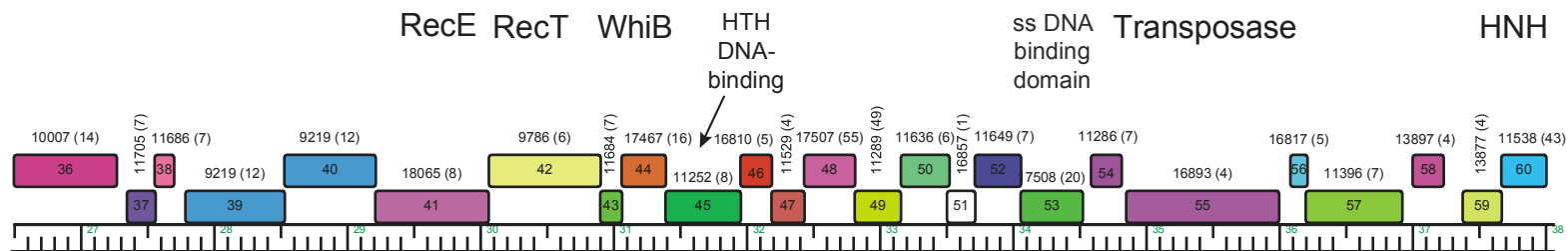

Supplement: Supplementary file 3 — Figure S2. Genome organization of phage E6. See Additional file 2: Figure S1 for details. (PDF 161 kb) [file 12866_2018_1159_MOESM3_ESM.pdf]

# G4

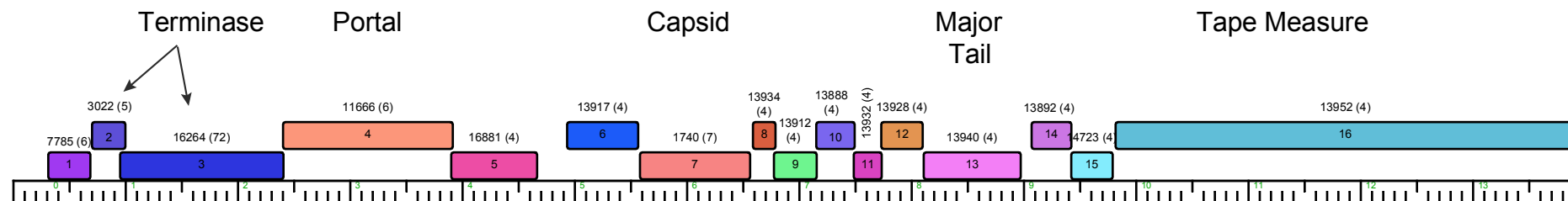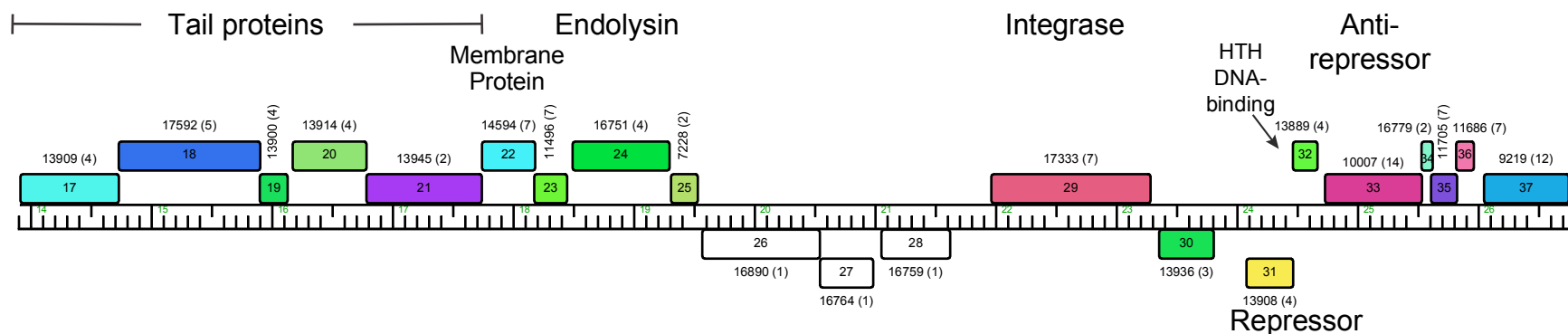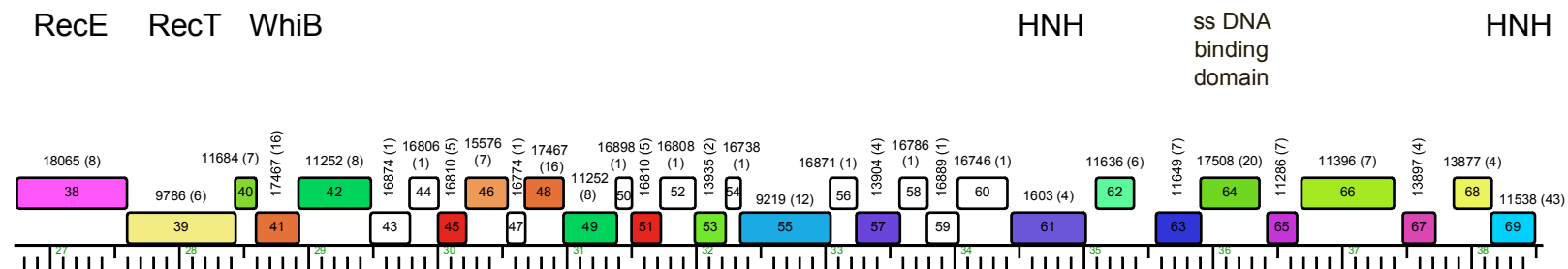

Supplement: Supplementary file 4 — Figure S3. Genome organization of phage G4. See Additional file 2: Figure S1 for details. (PDF 185 kb) [file 12866_2018_1159_MOESM4_ESM.pdf]

# E1

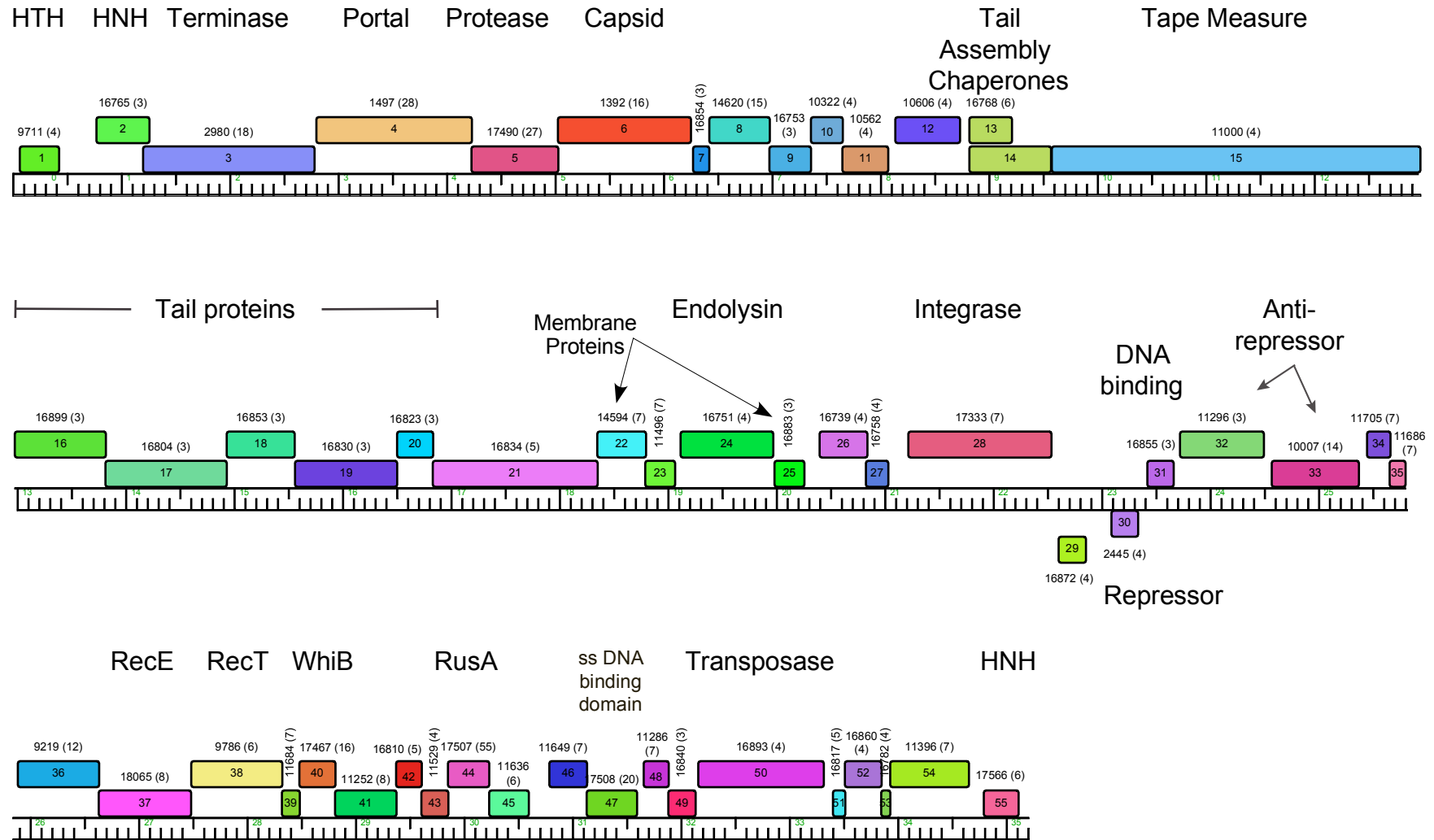

Supplement: Supplementary file 5 — Figure S4. Genome organization of phage E1. See Additional file 2: Figure S1 for details. (PDF 152 kb) [file 12866_2018_1159_MOESM5_ESM.pdf]

# B3

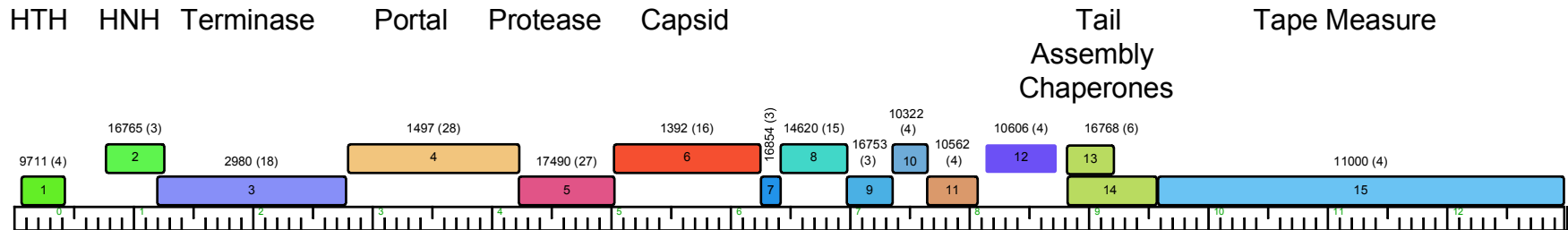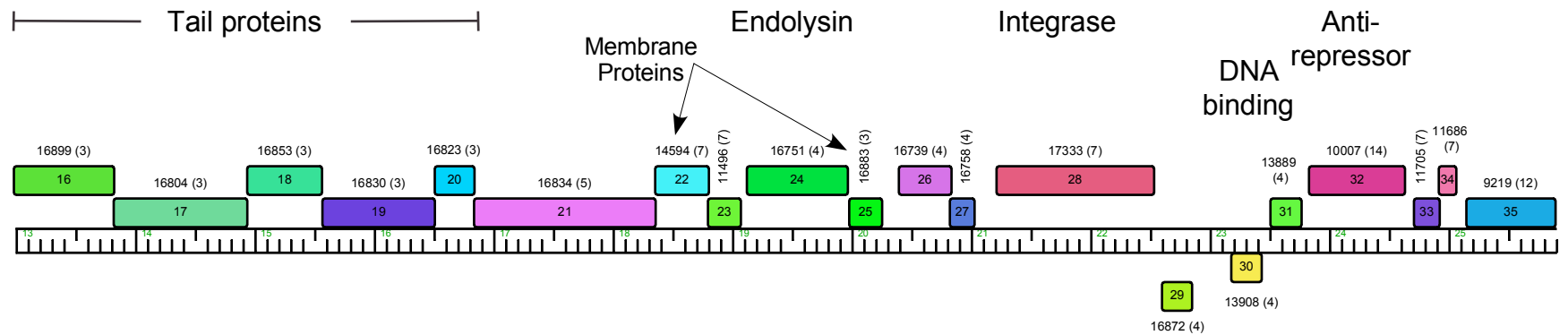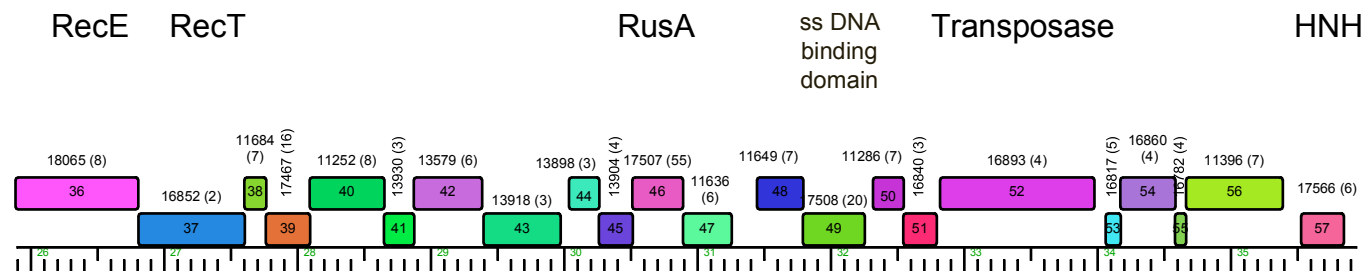

Supplement: Supplementary file 6 — Figure S5. Genome organization of phage B3. See Additional file 2: Figure S1 for details. (PDF 159 kb) [file 12866_2018_1159_MOESM6_ESM.pdf]

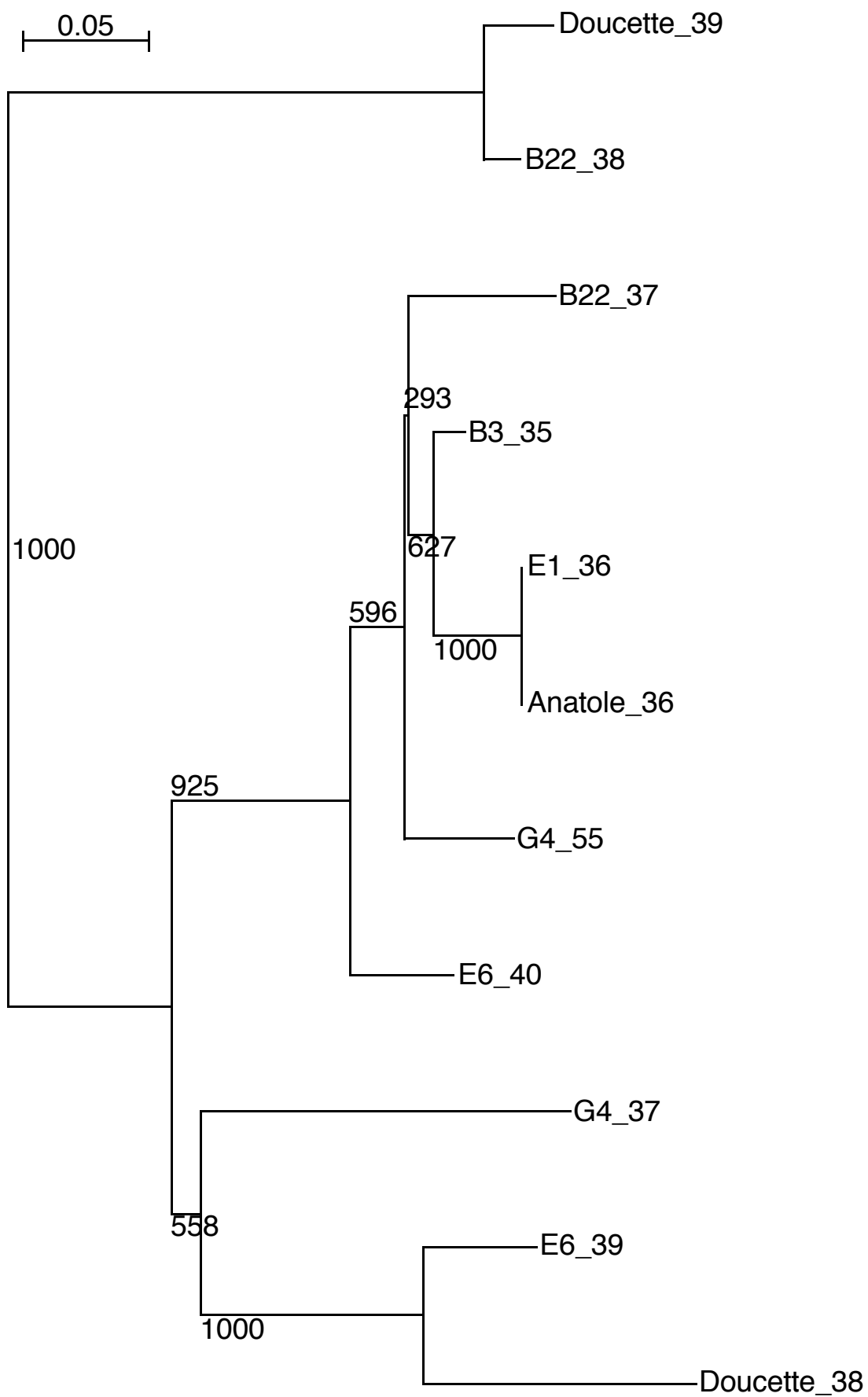

Supplement: Supplementary file 7 — Figure S6. Phylogenetic tree of tandemly repeated genes in P. freudenreichii phages. The four Cluster BW phages all encode two copies of related genes that are tandemly repeated in Doucette, B22, and E6, but which are separated by 17 genes in G4. The Cluster BV phage Anatole contains a single copy of this gene. Genes duplicated in one genome (e.g. Doucette 38 and 39) are more distantly related (51% aa identity) that with the corresponding gene in another genome (e.g. Doucette gp39 and B22 gp38 share 93% aa identity). Sequences were aligned using ClustalX and the tree drawn using NJPlot. Bootstrap values from 1000 iterations are shown. (PDF 22 kb) [file 12866_2018_1159_MOESM7_ESM.pdf]
